# Echocardiographic estimation of pulmonary capillary wedge pressure using the combination of diastolic annular and mitral inflow velocities

**DOI:** 10.1007/s12574-012-0142-0

**Published:** 2012-08-23

**Authors:** Tadafumi Sugimoto, Kaoru Dohi, Masaki Tanabe, Kiyotaka Watanabe, Emiyo Sugiura, Shiro Nakamori, Tomomi Yamada, Katsuya Onishi, Mashio Nakamura, Tsutomu Nobori, Masaaki Ito

**Affiliations:** 1 Department of Cardiology and Nephrology, Mie University Graduate School of Medicine, Tsu, Japan; 2 Department of Molecular and Laboratory Medicine, Mie University Graduate School of Medicine, 2-174 Edobashi, Tsu, 514-8507 Japan; 3 Department of Translational Medical Science, Mie University Graduate School of Medicine, Tsu, Japan; 4 Department of Clinical Cardiovascular Research, Mie University Graduate School of Medicine, Tsu, Japan

**Keywords:** Echocardiography, Displacement, Acute pulmonary embolism, Left ventricular function

## Abstract

**Background:**

We aimed to identify the clinical utility of a simple echocardiographic approach for estimating the pulmonary capillary wedge pressure (PCWP) on the basis of the combined assessment of mitral inflow and tissue Doppler mitral annular velocities.

**Methods:**

We retrospectively enrolled 165 patients who underwent both echocardiographic examination and right heart catheterization, and determined the diagnostic accuracy of echocardiography-derived parameters for estimating PCWP >18 mmHg.

**Results:**

Eighty-three patients had preserved left ventricular (LV) ejection fraction ≥50% (the PEF group) and 82 patients had reduced LVEF <50% (the REF group). The PEF group had higher peak early mitral annular velocity (*E*′) compared with the REF group. Eight patients in the PEF group but none in the REF group had normal LV diastolic function, represented as *E*′ >8 cm/s, and all of these patients had normal inflow pattern. The mean PCWP had the strongest correlation with the ratio of the peak early mitral inflow velocity (*E*) to the peak late diastolic mitral inflow velocity during atrial contraction (*E*/*A*) in both groups, followed by the left atrial diameter and *E*/*E*′ in both patient groups. Receiver operating characteristic (ROC) analysis demonstrated that the combination of abnormal *E*′ ≤8 and elevated *E*/*A* had high diagnostic accuracy compared with *E*/*E*′ in both patient groups with different cutoff values of *E*/*A* (1.81 in the PEF group and 1.16 in the REF group) for predicting mean PCWP >18 mmHg.

**Conclusion:**

After excluding patients with normal diastolic function using *E*′, conventional *E*/*A* is a reliable marker for predicting high PCWP and is superior to *E*/*E*′.

## Introduction

Invasively measured pulmonary capillary wedge pressure (PCWP) has been widely used as a surrogate for left ventricular (LV) filling pressure and is directly associated with functional capacity and prognosis in patients with heart failure [1–3]. Several echocardiography-derived parameters have been reported to provide non-invasive means for estimating the PCWP [4]. Current ultrasound systems have tissue Doppler presets for assessing mitral annular velocities [5] and the ratio of the peak early mitral inflow (*E*) velocity to the peak early mitral annular velocity (*E*′) is most widely used for estimating the PCWP in the clinical setting. However, several publications have recently raised concerns about the reliability of *E*/*E*′, especially in patients with preserved LV ejection fraction (EF), mainly because *E*′ is affected by LV morphology, regional function, and mitral annular structure, resulting in an erroneous PCWP estimation [6, 7].

Although the mitral inflow patterns, traditional, and simple Doppler indices have U-shaped relations to LV filling pressure, they can be used for the estimation of the PCWP with reasonable accuracy after discriminating patients with diastolic dysfunction from those having normal diastolic function and, thereby, normal inflow pattern. Although mitral annular *E*′ is influenced by multiple intrinsic factors as described above, it has a great ability to identify abnormal LV diastolic function. Therefore, a simple combination of *E*′ and mitral inflow pattern would provide a more precise estimation of high PCWP in patients having both reduced and preserved LVEF. Accordingly, our objective was to identify the potential reliability of a simple echocardiographic approach for predicting high LV filling pressure by the combined assessment of mitral inflow and tissue Doppler mitral annular velocities in patients with reduced and preserved LVEF with heterogeneous etiologies of heart disease.

## Materials and methods

### Study population

We retrospectively enrolled 373 consecutive patients ≥18 years of age who underwent both right heart catheterization and transthoracic echocardiography because of concerns about hemodynamic derangements at the Mie University Hospital between June 2004 and November 2011. From this group, we selected 165 patients after excluding patients with atrial fibrillation (*n* = 69), constrictive pericarditis (*n* = 3), pacemaker (*n* = 16), prosthetic valve (*n* = 25), hemodialysis (*n* = 14), cardiac shunt (*n* = 16), acute coronary syndrome (*n* = 21), and patients who exhibited worsening or improvement in New York Heart Association (NYHA) functional class during the brief time between the right heart catheterization and the echocardiography (*n* = 44). This study was approved for use by the Human Studies Subcommittee of Mie University Graduate School of Medicine.

### Right heart catheterization

Cardiac catheterization was performed in the resting supine position. A 6F balloon-tipped fluid-filled catheter was inserted via an introducer sheath placed in the right internal jugular vein for the measurement of cardiac output and intra-cardiac pressures, including the PCWP, and was connected to a physiologic pressure transducer with the zero level at the mid-axillary line. The wedge position was confirmed by fluoroscopy and pressure waveform, and the mean PCWP was measured at end-expiration.

### Echocardiography

All patients underwent routine transthoracic echocardiography using a Vivid 7 system (GE Vingmed, Horten, Norway) or an Aplio ultrasound system (Toshiba Medical Systems Corp., Tokyo, Japan) within 2 weeks (3 ± 4 days) before or after right heart catheterization. The size of the left atrium, interventricular and LV posterior wall thicknesses, LV end-diastolic and end-systolic diameter, and LVEF were assessed from the parasternal long-axis view [8]. Pulse-waved Doppler, using a sample volume placed at the tips of the mitral valve leaflets, was used to determine the mitral *E* velocity, peak late diastolic mitral inflow velocity during atrial contraction (*A* velocity), the ratio of *E* and *A* velocities (*E*/*A*), and the deceleration time (DT) of *E* velocity [5]. For tissue Doppler assessment, the sample volume was positioned at the septal sites of the mitral annulus [5]. *E*′ ≤8 was used as an indicator of LV diastolic dysfunction [5]. *E*/*E*′ was calculated for the prediction of a high mean PCWP. All Doppler values represent an average of 3 beats. The etiology and severity of valvular heart disease, if present, was identified by comprehensive echocardiographic assessments quantified by Doppler-derived echocardiography [9, 10]. We determined whether the combined use of *E*′ ≤8 and high *E*/*A* can more accurately predict elevated PCWP than *E*/*E*′-derived PCWP estimation under the clinical assumption that high *E*/*A* in the setting of abnormal LV diastolic function strongly indicates the presence of high filling pressure. Care was taken to obtain all images in the traditional imaging planes by an experienced echocardiographer. All images were evaluated blindly offline by an experienced sonographer at the Mie University Hospital echo core laboratory.

### Statistical analysis

Continuous variables were presented as the mean ± standard deviation (SD) and compared using the Student *t*-test or the Mann–Whitney *U*-test. Categorical variables were presented as percentage frequencies and differences between proportions were compared using the χ^2^ test. Receiver operating characteristic (ROC) curves were constructed to determine the optimal sensitivity and specificity for estimating PCWP >18 mmHg using inflow and tissue Doppler mitral annular parameters, and the differences in the area under the curve (AUC) between *E*/*E*′ and the combination of *E*′ and *E*/*A* were assessed by using Mann–Whitney *U* statistics. The diagnostic performance between *E*/*E*′ and the combination of *E*′ and *E*/*A* was compared with McNemar’s test. Confidence intervals were calculated according to the percentile method. A *p*-value <0.05 was accepted as being statistically significant. Data were analyzed using standard statistical software (SPSS version 19, Chicago, IL, USA).

## Results

### Patient characteristics

The basic demographic and clinical characteristics of the study participants are shown in Table 1. Of the 165 patients, 83 (50 %) patients had preserved LVEF equal to or greater than 50% (the PEF group) and 82 (50 %) patients had reduced LVEF lower than 50% (the REF group). Male gender was less frequent in the PEF group compared with the REF group. The PEF group consisted of a more heterogeneous population, including various valvular heart diseases. In contrast, 80 % of the REF group had either ischemic or non-ischemic dilated cardiomyopathy.

**Table 1:** Clinical characteristics of the study subjects

	All patients (*n* = 165)	PEF group (*n* = 83)	REF group (*n* = 82)
Mean age (years)	63 ± 14	65 ± 14	62 ± 13
Male gender [*n* (%)]	106 (64)	47 (57)	59 (72)*
Body mass index	24 ± 4	23 ± 3	24 ± 5
Etiology			
Ischemic heart disease [*n* (%)]	31 (19)	5 (6)	26 (32)*
Dilated cardiomyopathy [*n* (%)]	43 (27)	5 (6)	38 (48)*
Hypertensive heart disease [*n* (%)]	17 (10)	10 (12)	7 (9)
Hypertrophic cardiomyopathy [*n* (%)]	11 (7)	9 (11)	2 (2)*
Valvular heart diseases [*n* (%)]	43 (26)	39 (47)	4 (5)*
Aortic stenosis [*n* (%)]	25 (15)	22 (27)	3 (3)*
Aortic regurgitation [*n* (%)]	3 (2)	2 (2)	1 (1)
Mitral regurgitation [*n* (%)]	15 (9)	15 (18)	0 (0)*
Other [*n* (%)]	19 (12)	15 (18)	4 (5)*
Time interval between the two tests (days)	3 ± 4	3 ± 4	3 ± 4

*PEF* preserved ejection fraction, *REF* reduced ejection fraction

* *p* < 0.05 versus the PEF group

### Echocardiographic and hemodynamic measurements

The echocardiographic measurements are shown in Table 2. Adequate echocardiographic variables including mitral inflow and tissue Doppler signals were obtained in all patients. The PEF group had smaller left atrial diameter, thicker ventricular walls, and smaller LV diameter compared with the REF group. Although the mitral *E* velocity was similar in the two groups, the PEF group had lower *E*/*A* and longer DT compared with the REF group. The PEF group had higher *E*′ compared with the REF group, and 8 (10%) patients in the PEF group but none of the REF group had normal LV diastolic function, represented as *E*′ >8.

**Table 2:** Echocardiographic characteristics of the study subjects

	All (*n* = 165)	PEF group (*n* = 83)	REF group (*n* = 82)
Heart rate (beat/min)	72 ± 15	67 ± 12	76 ± 16*
Left atrial diameter (mm)	42 ± 7	41 ± 6	44 ± 8*
Interventricular wall thickness (mm)	11 ± 3	12 ± 4	10 ± 3*
LV posterior wall thickness (mm)	11 ± 3	12 ± 3	11 ± 2*
LV end-diastolic diameter (mm)	55 ± 11	48 ± 8	62 ± 8*
LV end-systolic diameter (mm)	41 ± 14	30 ± 6	53 ± 9*
LVEF (%)	49 ± 21	67 ± 9	30 ± 11*
Mitral *E* velocity (cm/s)	74 ± 30	75 ± 31	74 ± 30
Mitral *A* velocity (cm/s)	70 ± 29	75 ± 25	65 ± 31*
*E*/*A*	1.41 ± 1.21	1.16 ± 0.74	1.66 ± 1.51*
DT (ms)	194 ± 64	216 ± 65	170 ± 54*
*E*′ (cm/s)	4.6 ± 2.0	5.1 ± 2.4	4.1 ± 1.3*
*E*′ ≥8 cm/s [*n* (%)]	8 (5)	8 (10)	0 (0)*
*E*/*E*′	18.4 ± 10.3	17.1 ± 10.5	19.6 ± 9.9

*PEF* preserved ejection fraction, *REF* reduced ejection fraction, *LV* left ventricular, *Mitral E velocity* peak early diastolic mitral inflow velocity, *Mitral A velocity* peak late diastolic mitral inflow velocity during atrial contraction, *DT* deceleration time of the mitral *E* wave, *E*′ peak early mitral annular velocity

* *p* < 0.05 versus the PEF group

### Hemodynamic measurements

The hemodynamic measurements are shown in Table 3. Adequate hemodynamic variables were obtained in all patients. The PEF group had lower mean PCWP compared with the REF group, and the prevalence of patients having mean PCWP >18 mmHg was lower in the PEF group compared with the REF group. The cardiac index was higher in the PEF group than in the REF group.

**Table 3:** Results of the Swan–Ganz catheter

	All (*n* = 165)	PEF group (*n* = 83)	REF group (*n* = 82)
Heart rate (beat/min)	70 ± 14	66 ± 12	73 ± 14*
PCWP (mmHg)	13 ± 8	11 ± 7	14 ± 9*
PCWP >18 mmHg [*n* (%)]	37 (22)	8 (10)	29 (35)*
Systolic PAP (mmHg)	32 ± 14	30 ± 13	35 ± 15*
Mean PAP (mmHg)	20 ± 10	18 ± 9	23 ± 10*
Diastolic PAP (mmHg)	13 ± 7	11 ± 6	15 ± 8*
Mean RAP (mmHg)	5 ± 4	5 ± 3	6 ± 4
Cardiac index (L/min/m^2^)	2.8 ± 0.6	2.9 ± 0.6	2.6 ± 0.6*

*PEF* preserved ejection fraction, *REF* reduced ejection fraction, *PCWP* pulmonary capillary wedge pressure, *PAP* pulmonary artery pressure, *RAP* right atrial pressure

* *p* < 0.05 versus the PEF group

### Non-invasive estimation of PCWP >18 mmHg

**Fig. 1:**
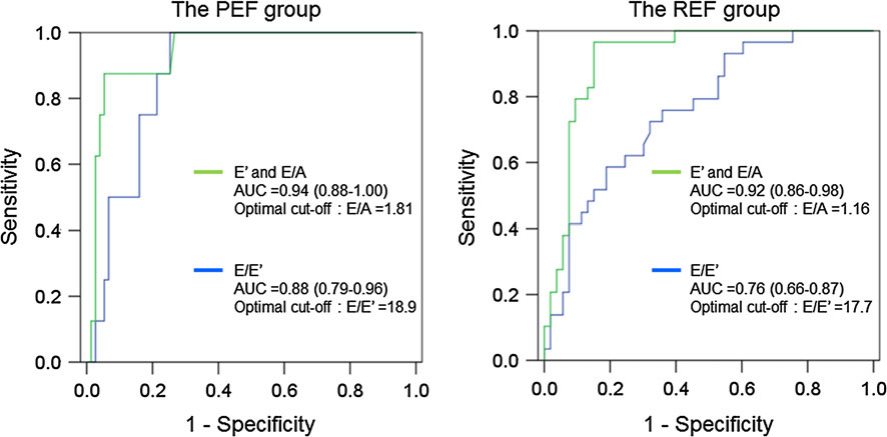
Receiver operating characteristic (ROC) curves to demonstrate the accuracy of the combination of abnormal *E*′ ≤8 and *E*/*A* and a single parameter of *E*/*E*′ for the prediction of mean PCWP >18 mmHg in the PEF group (*left*) and the REF group (*right*). *PEF* preserved ejection fraction, *REF* reduced ejection fraction, *AUC* area under the curve

**Fig. 2:**
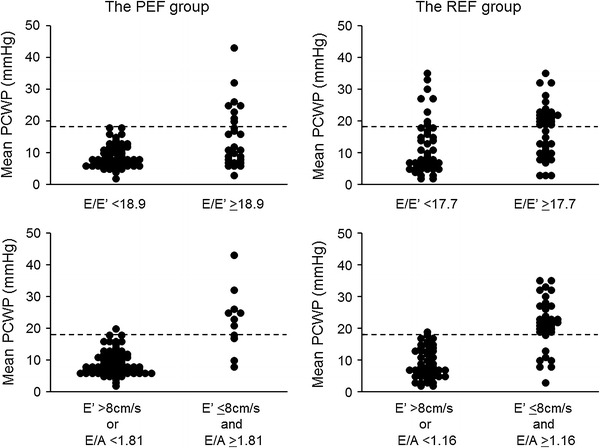
Mean PCWP versus groups defined by septal *E*/*E*′ (*top*) and the combination of *E*′ and *E*/*A* (*bottom*) in the PEF group (*left*) and the REF group (*right*). *PEF* preserved ejection fraction, *REF* reduced ejection fraction, *PCWP* pulmonary capillary wedge pressure

**Fig. 3:**
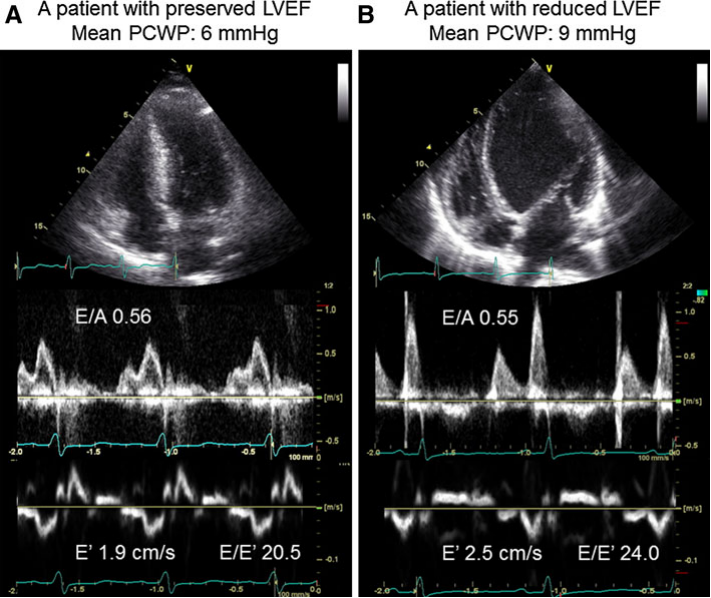
**a** Apical four-chamber view (*top*), mitral inflow (*middle*), and tissue Doppler-derived mitral annular velocity signals (*bottom*) in a patient with preserved LVEF and normal mean PCWP of 6 mmHg (*left*). **b** Apical four-chamber view (*top*), mitral inflow (*middle*), and tissue Doppler-derived mitral annular velocity signals (*bottom*) in a patient with reduced LVEF and normal mean PCWP of 9 mmHg (*right*). *LVEF* left ventricular ejection fraction, *PCWP* pulmonary capillary wedge pressure

Table 4 shows the correlations between the mean PCWP and echocardiographic measurements. The mean PCWP had the strongest correlation with *E*/*A*, followed by the left atrial diameter and *E*/*E*′ in both patient groups. All eight patients with normal *E*′ >8 cm/s in the PEF group had *E*/*A* >1.0 (1.83 ± 0.79). ROC analysis demonstrated that the combination of *E*′ ≤8 and high *E*/*A* had excellent diagnostic accuracy with large AUC in both patient groups with different cutoff values of *E*/*A* (1.81 in the PEF group and 1.16 in the REF group) for predicting mean PCWP >18 mmHg (Fig. 1; Table 5). Although the AUC of the combined *E*′ ≤8 and *E*/*A* ≥1.81 was comparable to that of *E*/*E*′ with a cutoff value of 18.9, its diagnostic accuracy was superior to *E*/*E*′, which had low positive predictive value (Fig. 1; Table 5) in the PEF group. The AUC of the combined *E*′ ≤8 and *E*/*A* ≥1.16 was larger and its diagnostic accuracy was higher for the prediction of PCWP >18 mmHg compared with those of *E*/*E*′ with a cutoff value of 17.7 in the REF group (Fig. 1; Table 5). There were 19 patients with false-positive results in the *E*/*E*′ estimation in the PEF group (Fig. 2), and they had similar *E*/*E*′ but lower *E* velocity and *E*′ compared with those patients with true-positive results (*E*/*E*′: 27.5 ± 15.0 vs. 25.4 ± 8.1, *p* = ns; *E* velocity: 78.6 ± 33.0 vs. 126.6 ± 27.4 cm/s, *p* < 0.05; *E*′: 3.1 ± 1.4 vs. 5.2 ± 1.1 cm/s, *p* < 0.05), indicating that patients with false-positive *E*/*E*′ results have very low *E*′ values, which generate disproportionally high *E*/*E*′, even in the setting of low LV filling pressure (Fig. 3a). Similar results were obtained in the comparison of mitral inflow and annular velocities between subjects with false-positive and true-positive *E*/*E*′ results (*E*/*E*′: 25.4 ± 8.1 vs. 29.2 ± 9.7, *p* = ns; *E* velocity: 77.2 ± 27.9 vs. 105.8 ± 23.2 cm/s, *p* < 0.05; *E*′: 3.1 ± 1.0 vs. 3.8 ± 0.9 cm/s, *p* < 0.05) in the REF group (Fig. 3b).

**Table 4:** Correlation of echocardiographic measurements with mean pulmonary capillary wedge pressure (PCWP)

	All (*n* = 165)	PEF group (*n* = 83)	REF group (*n* = 82)
Heart rate	0.10	0.21	−0.06
Left atrial diameter	0.47*	0.33*	0.50*
LVEF	−0.27*	−0.10	−0.20
*E*/*A*	0.59*	0.58*	0.59*
DT	−0.33*	−0.23*	−0.33*
*E*′	−0.02	0.06	0.02
*E*/*E*′	0.36*	0.28*	0.39*

*PEF* preserved ejection fraction, *REF* reduced ejection fraction, *LVEF* left ventricular ejection fraction, *DT* deceleration time of the mitral *E* wave, *E*′ peak early mitral annular velocity

* p < 0.05

**Table 5:** Accuracy of *E*/*E*′ versus the combination of *E*′ and *E*/*A* in the estimation of the mean PCWP

	*E*/*E*′	*E*′ and *E*/*A*	McNemar’s test (*p*-value)
PEF group	≥18.9	≤8 and ≥1.81	
Diagnostic accuracy	64/83 (77.1, [71.3–77.1])	78/83 (94.0, [88.3–95.9])	<0.05
Sensitivity	8/8 (100, [69.8–100])	7/8 (87.5, [57.9–97.7])	
Specificity	56/75 (74.7, [71.4–74.7])	71/75 (94.7, [91.5–95.8])	
Positive predictive value	8/27 (29.6, [20.7–29.6])	7/11 (63.6, [42.1–71.0])	
Negative predictive value	56/56 (100, [95.7–100])	71/72 (98.6, [95.3–99.7])	
REF group	≥17.7	≤8 and ≥1.16	
Diagnostic accuracy	57/82 (69.5, [59.3–77.6])	73/82 (89.0, [81.4–91.0])	<0.05
Sensitivity	21/29 (72.4, [58.0–83.8])	28/29 (96.6, [85.8–99.4])	
Specificity	36/53 (67.9, [60.0–74.2])	45/53 (84.9, [79.0–86.5])	
Positive predictive value	21/38 (55.3, [44.3–64.0])	28/36 (77.8, [69.1–80.1])	
Negative predictive value	36/44 (81.8, [72.3–89.3])	45/46 (97.8, [91.1–99.6])	

Values are expressed as number/total number and (% [95% confidence interval])

*PEF* preserved ejection fraction, *REF* reduced ejection fraction

## Discussion

We revealed the clinical utility of the combined use of *E*′ and mitral *E*/*A* and its superiority over *E*/*E*′ for predicting high LV filling pressure in patients with both reduced and preserved LVEF. ROC analysis demonstrated that the combination of abnormal *E*′ ≤8 and elevated *E*/*A* had high diagnostic accuracy compared with *E*/*E*′-derived PCWP estimation in both patients groups with different cutoff values of *E*/*A* (1.81 in the PEF group and 1.16 in the REF group) for predicting mean PCWP >18 mmHg.

The elevation of LV filling pressure is a unifying feature for heart failure, regardless of the underlying cause [11], and PCWP >18 mmHg has been recognized as one of the main therapeutic targets [12, 13]. Therefore, we employed a PCWP of 18 mmHg [13, 14], but not 12 or 15 mmHg [6, 7], as the cutoff value for evaluating the diagnostic accuracy of echocardiography-derived parameters for estimating high LV filling pressure in the present study. Several echo-Doppler parameters have been reported to provide non-invasive means for estimating the PCWP [4]. *E*′ or propagation velocity (*V*_p_) is a relatively preload-independent parameter representing LV relaxation, and, therefore, they have been shown to be useful for predicting elevated PCWP. Most of the current ultrasound systems have tissue Doppler presets for assessing mitral annular velocities [5], which are easy to obtain and in-depth data accumulated, so *E*/*E*′ is most widely used for estimating the PCWP in the clinical setting. However, there are several known limitations for the use of *E*/*E*′ in the estimation of LV filling pressure in various specific heart diseases or conditions, such as valvular heart disease and heart failure with preserved LVEF [6, 7]. Our data showed that *E*/*E*′ had high negative predictive value in patients with both preserved and reduced LVEF, but the positive predictive value was low, especially in patients with preserved LVEF. Although we should take into account the fact that the low prevalence of high mean PCWP might contribute to this low positive predictive value, the weak correlation between *E*/*E*′ and the mean PCWP indicates that high *E*/*E*′ is not a strong marker of high PCWP and the use of *E*/*E*′ in the estimation of LV filling pressure in patients with preserved LVEF is not recommended in the daily clinical setting. Patients with false-positive *E*/*E*′ estimation had similar *E*/*E*′ but had lower *E* velocity and *E*′ compared with those patients with true-positive results in patients with both preserved and reduced LVEF in the present study. These results indicate that patients with false-positive diagnosis on the basis of *E*/*E*′ results may have a very low *E*′ value, which generates disproportionally high *E*/*E*′, even in the setting of low LV filling pressure. Hay et al. investigated the effect of LV relaxation, as assessed by the exponential time constant of relaxation, on LV filling pressure in an experimental model [15]. They demonstrated that prolonged LV relaxation did not result in increases in LV filling pressure in the absence of volume loading. These results suggest that the worsening of LV relaxation alone is not sufficient for PCWP elevation, and, therefore, high *E*/*E*′ in the combination of very low *E*′ and non-elevated *E* velocity may not indicate high PCWP.

Because mitral inflow patterns are highly sensitive to preload and can change dramatically as LV filling pressure increases, the use of mitral valve inflow patterns for predicting high mean PCWP can be ideal after discriminating normal inflow patterns in patients with normal diastolic function. It has been well recognized that *E*′ ≤8 cm/s indicates impaired LV relaxation [5]. Although *E*′ is age-dependent [16] and, therefore, cutoff values of *E*′ for identifying diastolic dysfunction might vary between age groups, a single cutoff value of 8.0 was found to successfully identify patients with normal diastolic function and, hence, normal PCWP in the present study. The main diagnostic utility of this test lies in its high specificity and positive predictive value, making it a good rule out test for the exclusion of high LV filling pressure. We propose different *E*/*A* cutoff values for different patient populations for the prediction of mean PCWP >18 mmHg: 1.81 for patients with preserved LVEF and 1.16 for those with reduced LVEF. Small and stiff LV has the characteristic of being much more sensitive to preload changes compared with dilated LV [11]. Therefore, a given increase in LV filling pressure may generate higher early filling flow in patients with preserved LVEF than those with dilated LV, which resulted in the higher cutoff value of *E*/*A* for predicting the PCWP.

### Study limitations

Although we succeeded in the quantitative estimation of high LV filling pressure with the optimal cutoff by the combined assessment of mitral inflow and tissue Doppler mitral annular velocities, a potential limitation of the present study is the relatively small sample size with a low rate of high mean PCWP >18 mmHg in the PEF group, which lead to low positive predictive values of *E*/*E*′ estimation with wide confidence intervals. Another limitation of the present study is the heterogeneous etiologies of heart disease, especially in patients with preserved LVEF. Although we succeeded in reaching our primary point that showed statistically significant higher diagnostic accuracy and etiological varieties might bring favorable distribution in the mean PCWP, further study in larger populations is needed in order to specify the etiological differences that might contribute to the diagnostic performance of the combined assessment of mitral inflow and tissue Doppler mitral annular velocities in the prediction of high mean PCWP. We only measured septal mitral annular velocities, and neither diastolic function at the lateral mitral annulus nor the average of both annuli was assessed. However, septal *E*′ is considered to correlate well with LV diastolic function, and is widely used clinically [17]. Right heart catheterization and echocardiography were not measured simultaneously for estimating the PCWP, although great care was taken to obtain all measures in stable cardiac conditions for the evaluation of mitral inflow and the PCWP. No direct hemodynamic measurements of LV end-diastolic or left atrial pressure were performed [18]. Finally, other echocardiography-derived parameters such as the ratio of the systolic and diastolic velocities of the pulmonary venous inflow, systolic fraction of the pulmonary venous forward flow, and *V*_p_, which have been shown to be robust predictors of high LV filling pressures and cardiovascular mortality [19–21], were not included in the present study. These measures are limited by the inability to adequately image the pulmonary veins in some patients and by the limited reproducibility of *V*_p_.
